# Data-based selection of creep constitutive models for high-Cr heat-resistant steel

**DOI:** 10.1080/14686996.2020.1738268

**Published:** 2020-04-27

**Authors:** Hitoshi Izuno, Masahiko Demura, Masaaki Tabuchi, Yoh-ichi Mototake, Masato Okada

**Affiliations:** a Research and Services Division of Materials Data and Integrated System, National Institute for Materials Science, Ibaraki, Japan; b Research Center for Structural Materials, National Institute for Materials Science, Ibaraki, Japan; c The Institute of Statistical Mathematics, Tokyo, Japan; d Graduate School of Frontier Sciences, The University of Tokyo, Chiba, Japan

**Keywords:** Creep constitutive equation, grade 91 steel, bayesian free energy, model selection method, theta method, steady-state creep, 404 Materials informatics / Genomics, 106 Metallic materials

## Abstract

There are two types of creep constitutive equation, one with a steady-state term (steady-state type) and the other with no steady-state term (non-steady-state type). We applied the Bayesian inference framework in order to examine which type is supported by experimental creep curves for a Grade 91 (Gr.91) steel. The Bayesian free energy was significantly lower for the steady-state type under all the test conditions in the ranges of 50–90 MPa at 923 K, 90–160 MPa at 873 K and 170–240 MPa at 823 K, leading to the conclusion that the posterior probability was virtually 1.0. These findings mean that the experimental data supported the steady-state-type equation. The dependence of the evaluated steady-state creep rate on the applied stress indicates that there is a transition in the mechanism governing creep deformation around 120 MPa.

## Graphical Abstract

**Figure:**
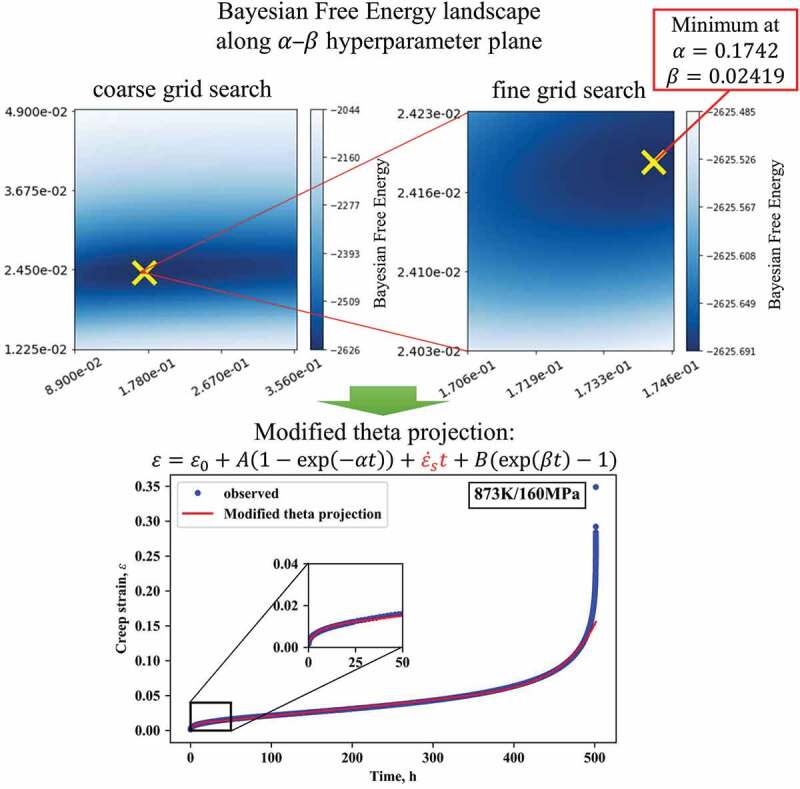

## 1. Introduction

Creep is a time-dependent phenomenon and is controlled by various mechanisms depending on the stage. In general, it starts at a relatively high rate followed by a decrease in the rate in the primary stage, and then the rate reaches a minimum and appears to become constant in the secondary stage. In the tertiary stage, the creep deformation gradually accelerates with time. The nature of the dominant mechanism in each stage can be characterized by the time dependence of the creep rate in most cases. For example, a decrease or increase in the creep rate is expected to indicate a change in the microstructure and/or in the amount and the structure of defects such as dislocations and voids. A constant creep rate, however, does not necessarily mean that the controlling microstructure is stable since an apparent steady state could occur when the decrease and increase in creep rate are balanced owing to the occurrence of both types of microstructural change. To examine the dominant mechanisms in the secondary stage, it is crucial to distinguish between the intrinsic and extrinsic mechanisms when a constant creep rate is observed.

In this study, we propose a Bayesian inference framework to judge whether there is an intrinsic steady state, on the basis of observed creep strain curves. Here, we reduce the problem to a model selection problem involving two types of creep constitutive equation, i.e. one with a steady-state term and the other with no steady-state term. If there is no steady state, the proposed framework should choose the equation with no steady-state term. For the non-steady-state model, we adopt the theta projection [1], which includes exponential-type deceleration and acceleration terms corresponding to the primary and tertiary stages, respectively. For the steady-state model, we add a linear time-dependent term, which was introduced in the modified theta projection [2]. In summary, the two creep constitutive equations are written as follows:

(1) $$\begin{array}{rl} & \varepsilon =\varepsilon_{0}+A\left(1-\exp\left(-\alpha t\right)\right)+{\dot{\varepsilon }}_{s}t+B\left(\exp\left(\beta t\right)-1\right)\\ & \;\;\;\;\;\;\;\;\;\;\;\;\left(modified\;theta\;projection,steady\text{-}state\;model\right) \end{array}$$

(2) $$\begin{array}{rl} & \varepsilon =\varepsilon_{0}+A\left(1-\exp\left(-\alpha t\right)\right)+B\left(\exp\left(\beta t\right)-1\right)\\ & \;\;\;\;\;\;\;\;\;\;\;\left(theta\;projection,non\text{-}steady\text{-}state\;model\right), \end{array}$$

where ε and t are the creep strain and test time, respectively. α and β are the time constants for the primary and tertiary stages, respectively, and $\varepsilon_{0}$, A, and B are constants with the dimension of strain. The constant ${\dot{\varepsilon }}_{s}$ in equation (1) corresponds to the steady-state creep rate in the secondary stage. For the model selection, we use the Bayesian inference framework. As detailed in the next section, we calculate, for each model, the Bayesian free energy which can be used as an indicator to evaluate which model is closer to the actual model that generated the observed data. To our best knowledge, there is no study to apply the Bayesian inference framework to the model selection problem in creep constituent equations.

The target material is one of the 9Cr-1Mo-Nb-V (Gr.91) steels, which are employed for high-temperature piping at thermal power plants owing to their excellent creep strength. Table 1 lists the previous studies in which the constitutive equation (1) or (2) was applied to the Gr.91 steel [2–4]. Both equations have been used and there is no consensus on whether the steady-state term is necessary.

**Table 1.** Some previous studies of creep curves in which the theta or modified theta method was applied to Gr.91 steel

Papers	Steady (modified theta)	Non-steady (theta or revised theta)	Other models	Test conditions	Minimum creep rate	Secondary (steady- state) creep
Factors influencing creep model equation selection Holdsworth et al. (2007) [2]	Applied	Not applied	–	–	–	–
Long-term creep characterization of Gr.91 steel by modified creep constitutive equations Kim et al. (2011) [3]	Not applied	Applied	Omega	873 K 130–200 MPa	$10^{-1}-10^{-4}$/h	Yes
Creep-rupture life prediction for 9Cr-1Mo-Nb-V weld metal Miyakita et al. (2015) [4]	Not applied	Applied	Omega	883 K 150 MPa	$3.85\times 10^{-5}$/h	Yes
				898 K 110–170 MPa	$3.25\times 10^{-3}$–$1.15\times 10^{-5}$/h	
				923 K 90–150 MPa	$6.10\times 10^{-3}$–$2.16\times 10^{-5}$/h	

## 2. Bayesian inference framework for model selection

In the Bayesian inference framework [5], the posterior probability of each model based on given data can be evaluated as follows: The posterior probability of a model $l=l\left(w_{l}\right)$ ($w_{l}$: parameter vector of model l) under given data D is considered. The posterior probability $P\left(l|D\right)$ for model l is given by

(3) $$p\left(l|D\right)=\frac{p(D|l)p\left(l\right)}{p\left(D\right)}$$

from Bayes’ theorem. On the other hand, let Λ be the set of all the considered models. $P\left(l|D\right)$ is evaluated as

(4) $$p\left(l|D\right)=\frac{p(l|D)}{\sum_{\lambda \in \Lambda }p(\lambda |D)}=\frac{\frac{p(D|l)p\left(l\right)}{p\left(D\right)}}{\sum_{\lambda \in \Lambda }\frac{p(D|\lambda )p\left(\lambda \right)}{p\left(D\right)}}=\frac{p(D|l)}{\sum_{\lambda \in \Lambda }P(D|\lambda )}$$

under the assumption that $p\left(l\right)$ is the same uniform distribution among all the considered models of Λ. p(D|l) is the model evidence and given by $p(D|l)=\int dw_{l}p(D|w_{l},l)\varphi (w_{l}|l)$, where $\varphi (w_{l}|l)$ is the prior distribution of parameters $w_{l}$ of model l. Let $F_{l}=-ln\left(p(D|l)\right)$. $F_{l}$ is called the Bayesian free energy of l [5,6]. Finally, $p\left(l|D\right)$ can be evaluated as

(5) $$p\left(l|D\right)=\frac{\exp\left(-F_{l}\right)}{\sum_{\lambda \in \Lambda }\exp\left(-F_{\lambda }\right)}.$$

Although the direct calculation of the Bayesian free energy (equation (4)) is difficult in general, it is possible for a linear model with variables having normal distributions. That is, let model *l* be a *c*-dimensional linear model$:\;y=\sum_{i=1}^{c}w_{i}x_{i}\;\left(w_{l}=\left\{w_{i}\right\}\right)$, and the number of data be *N*. If both $p(D|w_{l},l)$ and $\varphi (w_{l}|l)$ are multivariate normal distributions of dimensions *N* and *c* with variances $\sigma^{2}$ and $\sigma_{1}^{2}$, respectively, the Bayesian free energy is given by

(6) $$\begin{array}{rl} & F\left(\sigma^{2},\sigma_{1}^{2}\right)=-ln\left(\int dw_{l}{\left(\frac{1}{2\pi \sigma^{2}}\right)}^{\frac{N}{2}}\exp\left(-\frac{1}{2\sigma^{2}}\left(y^{T}-{w_{l}}^{T}X\right){\left(y^{T}-{w_{l}}^{T}X\right)}^{T}\right){\left(\frac{1}{2\pi \sigma_{1}^{2}}\right)}^{\frac{c}{2}}\exp\left(-\frac{1}{2\sigma_{1}^{2}}{w_{l}}^{T}w_{l}\right)\right) \end{array}$$

where $X=\left[{\left\{x_{i}\right\}}_{j}\right]$ and $y=\left\{y_{j}\right\}$ meaning given data are a c×N matrix of the explanatory variables and an *N*-dimensional vector of the objective variable, respectively, and the superscript *T* means the transpose of vector.

This integration can be performed directly by Gaussian integration. The first exponential term corresponds to the bias of quadratic errors between the model and data, and minimizing this term may cause the overfitting problem. However, there is also a second exponential term for evaluating the complexity of the model. Thus, minimizing the Bayesian free energy is a robust way of optimizing the model parameters since it is equipped with a mechanism to avoid the overfitting problem [5].

The question is how to reduce the nonlinear creep constitutive equations into linear problems. Here, we treat the exponential terms as constant and hyperparameterize the time constants α and β. That is, by letting $R\left(\alpha ,t\right)=1-\exp\left(-\alpha t\right)$ and $S\left(\beta ,t\right)=\exp\left(\beta t\right)-1$, and applying R and S to equations (1) and (2), they are reduced to the following linear functions:

(1) $$\varepsilon =\varepsilon_{0}+AR+{\dot{\varepsilon }}_{s}t+BS$$

(2) $$\varepsilon =\varepsilon_{0}+AR+BS.$$

For these linear functions, we can calculate the Bayesian free energy by applying equation (6). Then, we optimize the hyperparameters α and β so that the Bayesian free energy is minimized.

**Figure 1.**
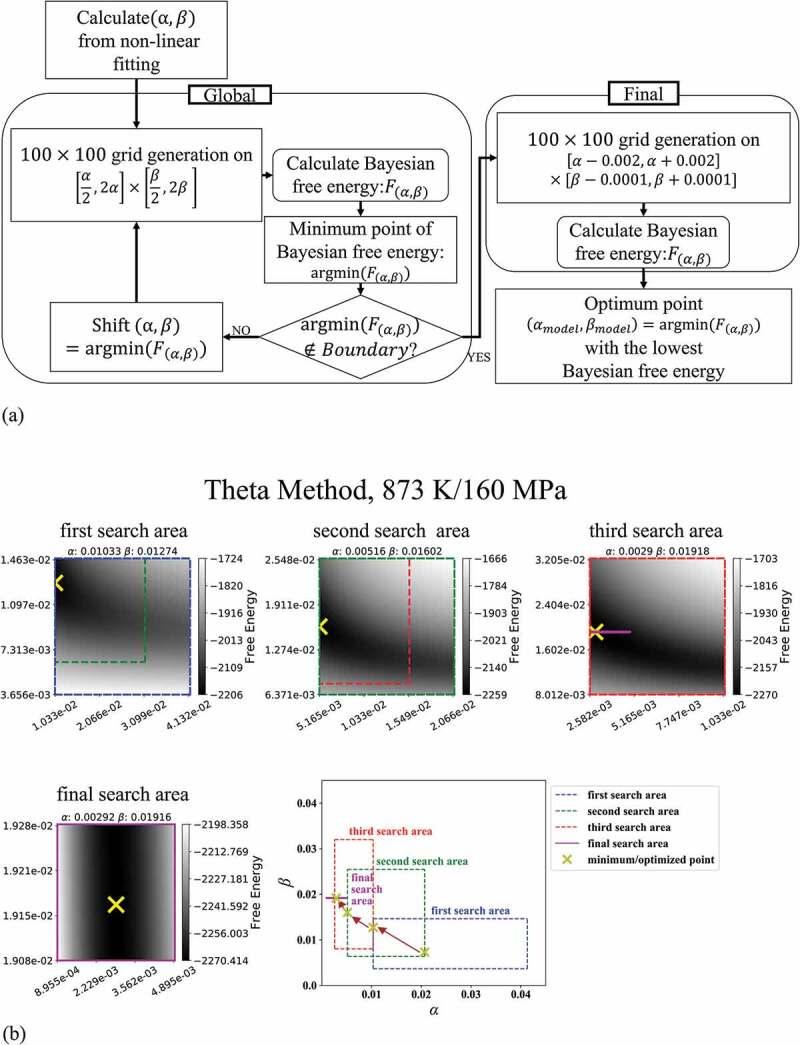
(a) Schematic diagram of model selection method with grid search. (b) shows how our algorithm worked in the case of applying the theta method to the creep strain curve for 873 K/160 MPa. The four heat maps show the distribution of Bayesian free energy by greyscale, where the point of the lowest free energy in each search area is indicated by the yellow cross mark. The green, red, and purple rectangles inserted in the top three maps show the next search area, respectively. The right-bottom plot shows the transition from the first search area to the final search area

Figure 1(a) shows the algorithm for optimizing α and β to minimize the Bayesian free energy based on a grid search method. Firstly, we performed a conventional nonlinear fitting to the experimental data to obtain initial guesses of α and β, respectively. Around the initial guesses, we then defined the first search area within the range from double to half of them, setting the number of grids to 100 for each parameter. We calculated the Bayesian free energy at each grid point and determined the point that exhibited the lowest Bayesian free energy within the search area. When the lowest free energy point was at the edge of the search area, we redefined a new search area around the point in the same way as we defined the first search area, that is, we set it within the range from double to half of the value of the point. This is because there might be a point with a lower value outside the previous search area. We continued the same search process until we found the lowest free energy point inside the search area. Then, we performed the final optimization step, for which we set the search area within the range from α−0.002 to α+0.002 and β−0.0001 to β+0.0001 throughout all the creep test conditions. The final optimization step enabled us to obtain the optimized values with uniform accuracy for all creep test conditions. Figure 1(b) shows how our algorithm worked in the case of applying the theta method to the creep strain curve for 873 K/160 MPa. In the search, we assumed that α and β were positive from the characteristics of the experimental creep curves. The computational calculations were performed on Python 3.5.2/NumPy 1.13.1/SciPy 0.19.0/scikit-learn 0.18.1 on Ubuntu 16.04 with a Precision Tower 7910 (Dell, USA).

## 3. Experimental procedures

Part of the creep results used in the study was previously reported [7], namely, most of the rupture time data except for those at 823 K/170 MPa, 873 K/90 MPa, and 923 K/50 MPa, and three creep strain curves at 873 K/160, 140, and 130 MPa. The procedures for the creep tests and the sample preparation were also given in the previous work [7]. The chemical composition of the Gr.91 steel used in this study is summarized in Table 2. We conducted creep tests under several constant loads at temperatures of 823 K, 873 K, and 923 K. Specimens with a gauge of 6 mm diameter and 30 mm length were cut from a plate. The nominal strain was obtained from the measured displacement normalized by the initial gauge length. For the analysis, the period from the beginning of the creep test up to 98% of the total rupture time was used to exclude the extrinsic increase in the creep rate attributable to the significant increase in true stress with the reduction of the cross section.

**Table 2.** Chemical composition of the 9Cr-1Mo-Nb-V (Gr.91) steel

	C	Si	Mn	P	S	Cu	Ni	Cr	Mo	V	Nb	Al	N
(mass %)	0.10	0.25	0.43	0.006	0.002	0.012	0.06	8.87	0.93	0.19	0.07	0.014	0.06

## 4. Results and discussion

Table 3 lists the creep test conditions, observed rupture time, and minimum creep rate. The creep rupture times and minimum creep rates obtained in this study were within the same ranges as those obtained in previously reported studies [2–4], and the consistency in the range of minimum creep rates can be partly confirmed by comparing Tables 1 and 3. The minimum creep rate and the creep rupture time in each creep test have an inversely proportional relationship.

**Table 3.** Observed rupture time and creep rate, Bayesian free energy, posterior probability of the two models, ratio of fitting parameters α for the modified theta method ($\alpha_{Mod\theta }$) and theta method ($\alpha_{\theta }$), and the ratio of the steady-state region to the rupture time for the modified theta method in the present ASME Gr.91 steel creep tests. Values for the Bayesian free energy are rounded to integers. Values for the posterior probability, the ratio of fitting parameters α, and the ratio of the steady-state region are rounded to three significant figures

Test conditions				Free energy			Posterior probability			
Temperature (K)	Stress (MPa)	Rupture (h)	Minimum creep rate(1/h)	Modified Theta	Theta	Modified theta		Theta	$\frac{\alpha_{\theta }}{\alpha_{Mod\theta }}$	Steady-state region ratio
823	240	290.7	1.76$\times 10^{-4}$	−2322	−2002	1		0	0.0359	0.563
	220	2392.5	1.05$\times 10^{-5}$	−2980	−2473	1		0	0.0646	0.709
	200	10,432.9	3.58$\times 10^{-6}$	−3883	−3370	1		0	0.0546	0.585
	190	18,514.7	1.44$\times 10^{-6}$	−11,016	−9453	1		0	0.0651	0.554
	170	47,104.5	3.46$\times 10^{-7}$	−24,936	−22,427	1		0	0.00813	0.500
873	160	501.4	9.65$\times 10^{-5}$	−2625	−2270	1		0	0.0167	0.579
	140	2550.2	1.48$\times 10^{-5}$	−3992	−3432	1		0	0.0271	0.579
	130	6036.8	5.33$\times 10^{-6}$	−2826	−2449	1		0	0.0232	0.483
	110	19,907.0	9.60$\times 10^{-7}$	−11,258	−10,693	1		0	0.00107	0.419
	100	40,307.4	5.48$\times 10^{-7}$	−12,410	−11,586	1		0	0.00107	0.503
923	90	73,960.9	2.83$\times 10^{-7}$	−9612	−9585	1		0	0.00124	0.342
	90	928.0	3.50$\times 10^{-5}$	−3141	−3026	1		0	0.000102	0.469
	80	2726.1	1.18$\times 10^{-5}$	−2757	−2688	1		0	0.0000820	0.460
	70	8385.6	4.75$\times 10^{-6}$	−3537	−3428	1		0	0.000129	0.382
	50	60,181.2	6.19$\times 10^{-7}$	−16,654	−15,000	1		0	0.0159	0.618

**Figure 2.**
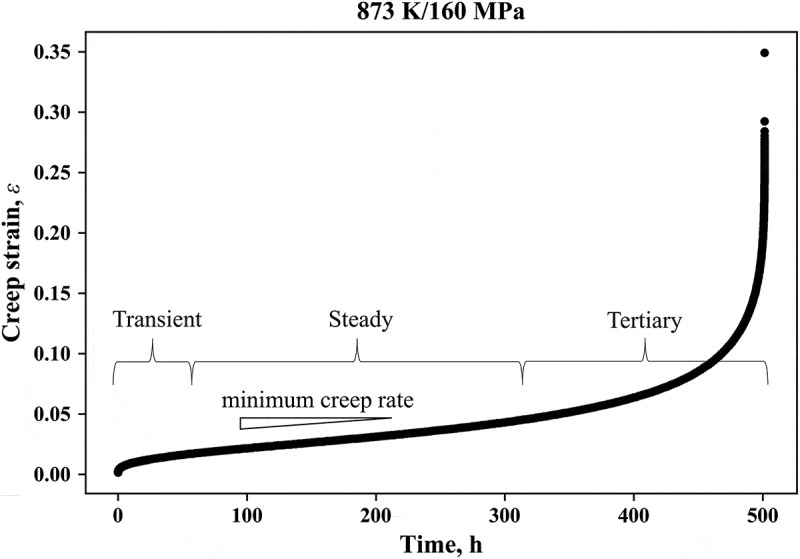
Creep strain curve of Gr.91 steel at 873 K/160 MPa

Figure 2 shows the creep strain curve at 873 K/160 MPa. The shape of the curve was upper convex in the primary stage from the start of the test to 20 h, showing a monotonic decrease in the creep rate. Then, it became almost a straight line from 20 to 300 h in the secondary stage, indicating that the creep rate was constant. In the tertiary stage after 300 h, the curve increased gradually, showing the increase in creep rate, and finally, the specimen ruptured at 501.4 h. All the creep strain curves obtained in this study had these three stages. The minimum creep rates listed in Table 3 were obtained from the gradient of the linear part of the creep strain curves in the second stage.

Using the obtained creep strain curves, we calculated the Bayesian free energies for the two types of constitutive equation, i.e. the modified theta projection (equation (1)) and the theta projection (equation (2)). Table 3 summarizes the results. Under all the test conditions, the modified theta projection, i.e. the steady-state model, exhibited lower Bayesian free energies than the theta projection, i.e. the non-steady-state model. This means that the experimental data supported the steady-state model rather than the non-steady-state model. We also calculated the posterior probability from the Bayesian free energy. Here, we explain the actual calculation in the case of 873 K/90 MPa, where the difference in Bayesian free energy is the smallest among all the test conditions. The Bayesian free energies were −9612 and −9585 for the modified theta and theta projection methods, respectively. The posterior probability for each method can be calculated from equation (5) with the set of models $\Lambda =\left\{modified\text{-}theta,theta\right\}$ as follows:

(7) $$P\left(modified\;theta|873K/90MPa\right)=\frac{\exp\left(-9612\right)}{\exp\left(-9612\right)+\exp\left(-9585\right)}=0.999999999998$$

and

(8) $$P\left(theta|873K/90MPa\right)=\frac{\exp\left(-9585\right)}{\exp\left(-9612\right)+\exp\left(-9585\right)}=1.87952881654\times 10^{-12}.$$

Although the difference in the Bayesian free energy is only 27, the posterior probability is clearly higher for the modified theta projection and is virtually one. As shown in Table 3, we confirmed that the posterior probabilities of the modified theta projection and theta projection are virtually one and zero under all the test conditions, respectively. Consequently, a model assuming a steady-state term was explicitly supported by the creep data obtained from the Gr.91 steel.

**Figure 3.**
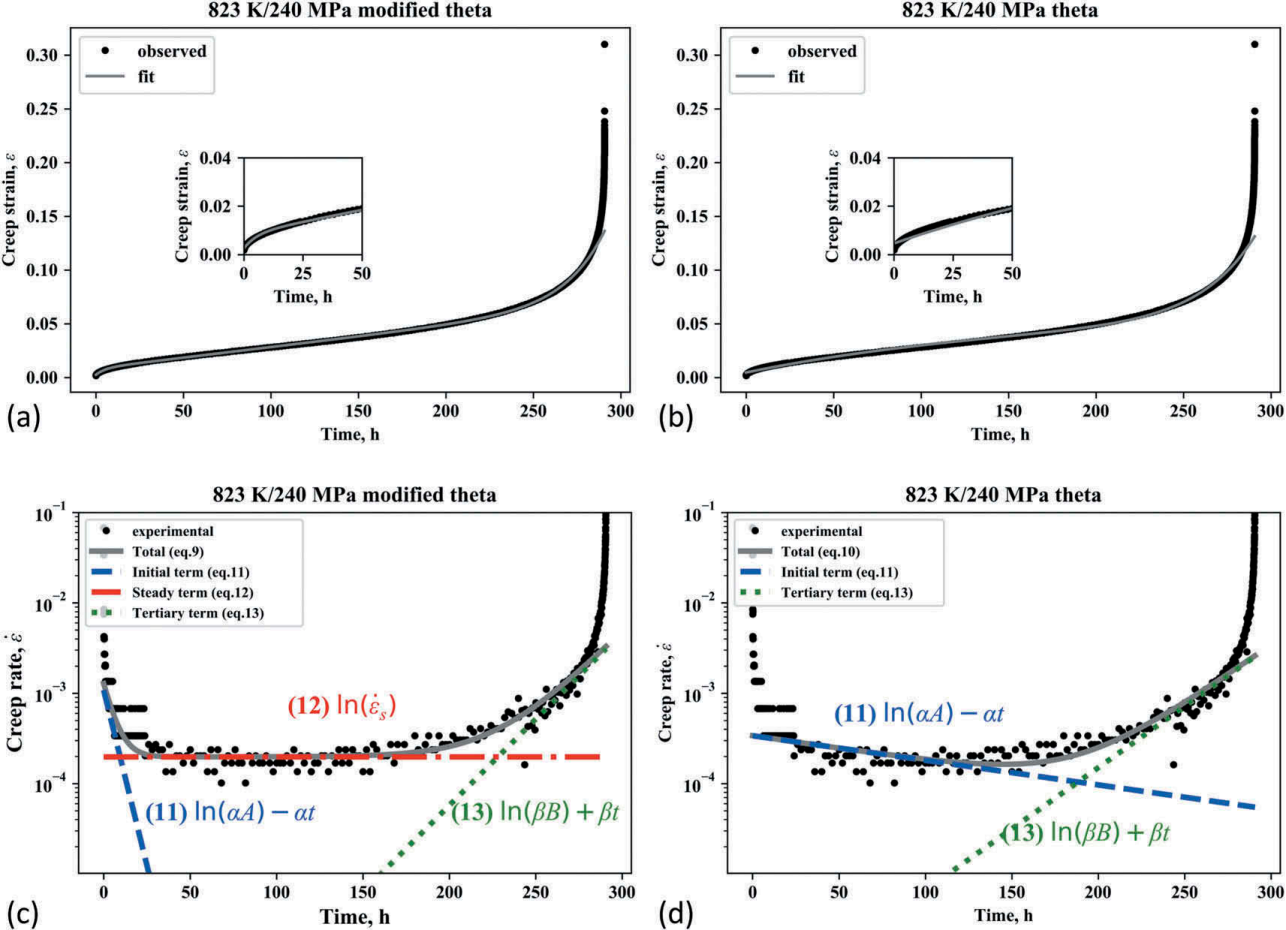
Fitting of Gr.91 steel creep curve at 823 K/240 MPa with (a) modified theta method and (b) theta method. Log of the creep strain rate against time for 823 K/240 MPa fitted with (c) modified theta method and (d) theta method. The insets in (a) and (b) are enlargements of the primary region. The formulae in (c) and (d) are the derivatives of each term of equations (1) and (2)

Figure 3(a,b) show the experimental creep strain curves and those calculated with the parameters at the minima of the Bayesian free energies for the modified theta and theta projections, respectively, for 823 K/240 MPa. The experimental curves (black dots) were well reproduced by the calculated ones (gray lines) for both projections. In detail, the theta projection method exhibited some discrepancy, especially in the primary region up to 50 h (see the inset of Figure 3(b)). In contrast, the modified theta projection method followed the experimental curves very well even in the primary stage, as shown by the inset of Figure 3(a).

The difference in the reproducibility of the creep strain curve is more clearly depicted in the creep strain rate curves shown in Figure 3(c, d), which are plots of the log of the creep strain rate against time. For more detailed analysis, we decompose the terms in each equation. The derivatives of equations (1) and (2) with respect to time t are

(9) $$\dot{\varepsilon }=A\alpha \exp\left(-\alpha t\right)+{\dot{\varepsilon }}_{s}+B\beta \exp\left(\beta t\right)$$

(10) $$\dot{\varepsilon }=A\alpha \exp\left(-\alpha t\right)+B\beta \exp\left(\beta t\right).$$

In the plot of log $\dot{\varepsilon }$ versus t, the primary, steady-state, and tertiary creep terms are asymptotic to the lines

(11) $$ln\left({\dot{\varepsilon }}_{primary}\right)=ln\left(A\alpha \exp\left(-\alpha t\right)\right)=\ln\left(\alpha A\right)-\alpha t$$

(12) $$ln\left({\dot{\varepsilon }}_{secondary}\right)=\ln\left({\dot{\varepsilon }}_{s}\right)$$

(13) $$ln\left({\dot{\varepsilon }}_{tertiary}\right)=\ln\left(B\beta \exp\left(\beta t\right)\right)=\ln\left(\beta B\right)+\beta t,$$

respectively. In Figure 3(c, d), we drew the lines given by equations (11) – (13) for the modified theta projection method (Figure 3(c)) and the lines given by equations (11) and (13) for the theta projection method (Figure 3(d)). The modified theta projection method traced the changes in the creep strain rate well in the whole region, i.e. the extensive drop in the primary stage, the steady state in the secondary stage, and the gradual increase in the tertiary stage. On the other hand, the theta projection method failed to follow the extensive drop in the primary stage and showed a very gradual decrease in this stage owing to the trend in both the primary and secondary regions being represented by a single average line. It turns out that the modified theta model well captured the gradient of the extensive drop in the primary stage (blue broken line) and that of the gradual increase in the tertiary stage (green dotted line). In addition, note that most of the secondary stage is governed by the steady-state term (equation (12)) as shown by the red dot-dashed line. That is, the contribution of the other time-dependent terms (equations (11) and (13)) can be regarded as negligible in the secondary stage. This implies that there is a steady state for a certain period of time in the creep deformation. On the other hand, for the theta projection method, no steady state appears, so one can expect an apparent steady state by compensating for the decrease in the primary stage and the increase in the tertiary stage. In practice, as shown in Figure 3(d), the deceleration rate was underestimated in the theta projection method since it tended to represent both the primary and secondary stages by a single deceleration term (equation (11)). Table 3 lists the ratio of the time constant α, indicating the deceleration rate, for the modified theta and theta projection models. The ratio clearly shows that the theta projection method significantly underestimated the rate of decrease compared with the modified theta projection method.

**Figure 4.**
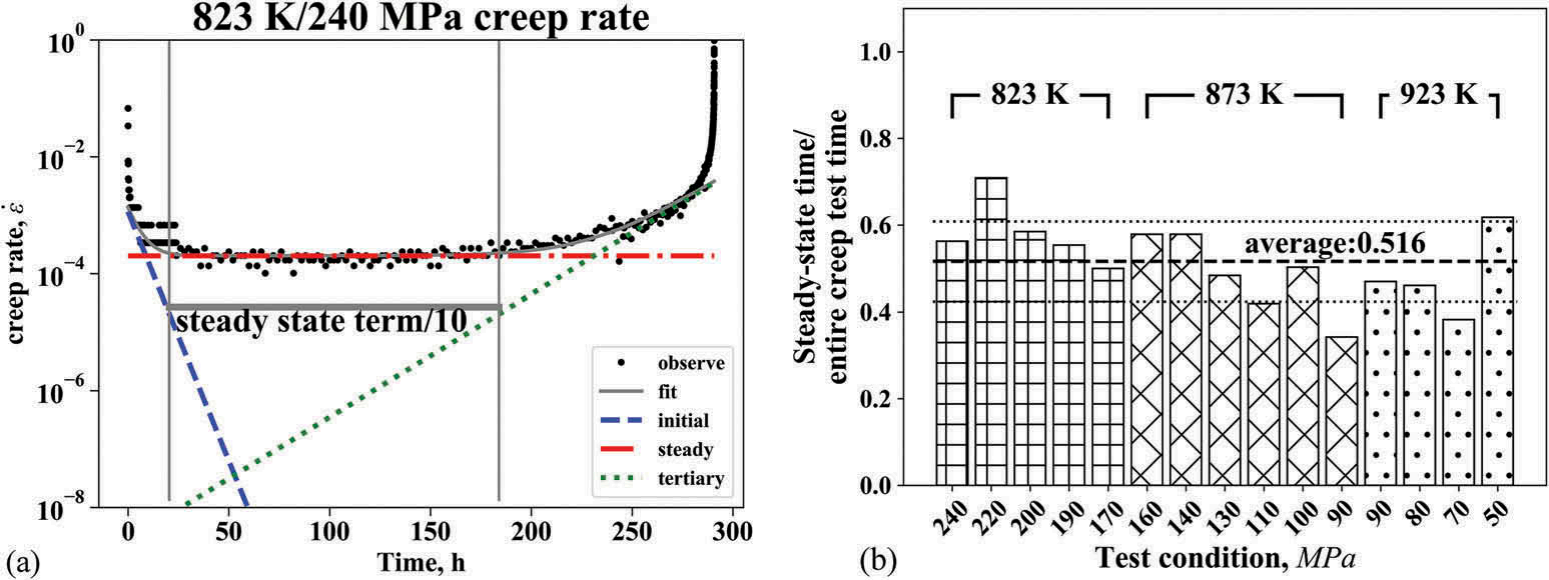
(a) Schematic figure of log of the creep strain rate against time with its fitting by modified theta method. Blue broken, red dot-dashed, and green dotted lines are the asymptotes of the corresponding terms of the equation (similar to FIG. 3.) The gray horizontal line shows the region where the steady-state term (equation (12)) is more than 10 times larger than the other terms (equations (11) and (13)), (b) Steady-state length ratio of Gr.91 steel creep curve estimated by modified theta method. Each length is normalized by the rupture time of the creep test

We evaluated the time of the steady-state region as a fraction of the entire creep rupture time, as schematically shown in Figure 4(a). Here, the steady-state region was defined as the region where the magnitude of the steady-state term is at least 10 times larger than that of the primary or tertiary term with respect to the creep rate. The steady-state region at 823 K/240 MPa is identified as being between two solid gray vertical lines in Figure 4(a). In the same way, the steady-state regions were identified for the other test conditions (see Figure S1 in the supplement material). Figure 4(b) shows the time fraction of the steady-state region versus the creep life under various testing conditions. The average fraction of all test results is 0.516 and the standard deviation is 0.0923, indicating that almost 50% of the creep deformation is governed by a mechanism leading to the steady state for the Gr.91 steel.

The present conclusion supports a widely used approximation named Norton’s law in the numerical simulation of creep deformation behavior [8]. The Norton’s law assumes a constant creep rate obeying the power law of the applied stress [9,10]. To our best knowledge, there was so far no report confirming the validity of the assumption of constant creep rate using a rational method based on creep curves. The present Bayesian inference method revealed that it is reasonable to assume a constant creep rate at least up to 50% of creep life for the Gr.91 steel.

**Figure 5.**
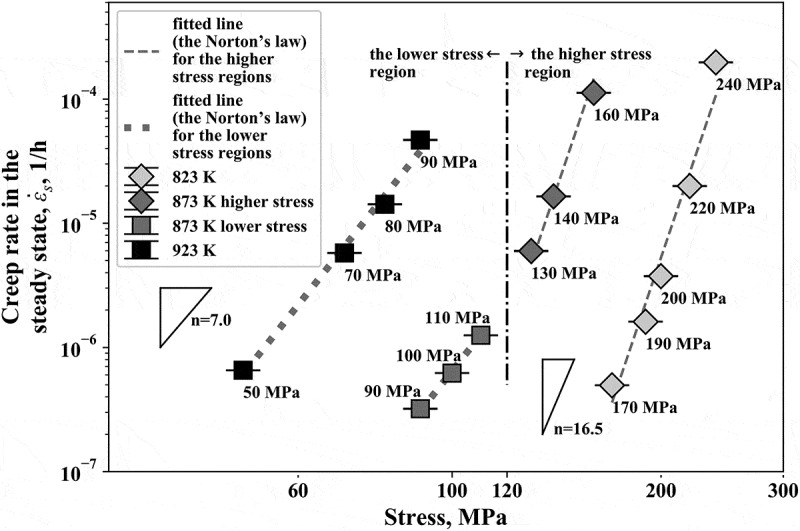
Double logarithmic plot of the creep rate in the steady state, ${\dot{\varepsilon }}_{s}$ versus applied stress. The standard deviation of the obtained posterior probability of each creep rate is given by the error bars

The power factor in the Norton’s law is related to the dominant mechanism of the creep deformation and it is represented by the gradient of a linear curve in the double logarithmic plot of the creep rate in the steady state, ${\dot{\varepsilon }}_{s}$ versus the applied stress. Figure 5 plots the double logarithmic plot of the steady-state creep rate versus the applied stress. The standard deviation of the obtained posterior probability of each rate is shown by the error bar, though the magnitude is so small, 10^−7^ h^−1^ order at most, that the error bars are not visible at all. As shown in Figure 5, there was a bent of the curve at a stress of 120 MPa at 873 K, indicating the change in the mechanism with respect to the applied stress. Let us divide the stress region below and above 120 MPa; thus, each data points at 923 K and 823 K belongs to the lower and higher stress region, respectively. It turned out that all the data points at 923 K can be fitted to a linear curve with the same gradient as that for the fitting line in the lower stress region at 873 K. Similarly, the fitting curve at 823 K had the same gradient as that in the higher stress region at 873 K. Thus, there were at least two regions having each mechanism in terms of the applied stress, irrespective of temperature. The Gr.91 steel exhibits long-term stability of the martensitic structure owing to fine precipitates introduced by tempering [11]. According to previous works [12–14], however, the martensitic structure is not necessarily stable and the lath boundary of the martensitic structure migrates under an applied stress in creep deformation. For example, Endo et al. [14] claimed that the dislocations composing the subgrain boundary like the lath boundary, which are pinned by the fine precipitates, can migrate by unpinning under an applied stress above a certain level and estimated that the stress necessary for unpinning is approximately 100 MPa. As mentioned in the former paragraph, our analysis suggested that the mechanism leading to the steady state is shifted around almost the same stress level. The agreement of the critical stress level indicates that the mechanism governing the steady state is strongly related to the stability of the martensitic structure.

Then, we separately discuss the possible mechanism in the two regions in terms of the applied stress below and above ~120 MPa. In the lower stress region, the work by Endo et al. [14] suggests that the coarsening mechanism by the applied stress was not activated and we thus consider that the martensitic structure should be stabilized by the precipitates, leading to the steady state of creep deformation. While the coarsening of the fine precipitates progressively occurs, there is a certain duration of the steady state; this is because there should be a sufficient amount of fine precipitates in the initial microstructure. In fact, Armaki et al. [15] experimentally showed that there is an incubation time to initiate the subgrain growth in high-Cr ferritic steels and that under a relatively low applied stress the incubation time is reasonably long, compatible to that observed at static recovery condition without applied stress. Thus, the steady state is supposed to end when the stabilization effect by the precipitates is lost by their coarsening. On the other hand, the martensitic structure is not stable in the higher stress region [14]. A possible mechanism here is that the migration of the subgrain boundary by unpinning from the precipitates dominates the creep deformation. Considering a high density of the subgrain boundaries in the Gr.91 steel, it is likely that their migration can yield most of the creep deformation, though the quantitative confirmation is out of the scope of the present work. Consequently, it is assumed that the steady state is controlled by the unpinning of the subgrain boundary from the precipitates.

Lastly, we would like to discuss about a limitation in the present model selection. The model selection result depends not only on the data but also on the set of targeted models. That is, it is impossible to conclude that the modified theta projection is the best among all the possible creep constitutive equations. There are several sophisticated constitutive equations including more terms and thus it would be helpful to apply this framework to these equations. Since the present model selection framework used the hyperparameterization to reduce the non-linear equations into linear problem, it would be necessary to modify the framework if applying it to more complicated constitutive equations including too many non-linear terms. This modification will be a future work. In spite of the limitation of the present model selection, we still consider that our analyses totally demonstrated the appropriateness of assuming the steady state for the present Gr.91 steel. In fact, the period of the steady state was significantly long, over 50% of the creep deformation in average for all the test conditions (Figure 4(b)), when the modified theta projection was assumed. Furthermore, the creep rate in the steady state was well fit by a simple power low of the applied stress, the Norton law, assuming two regions (Figure 5). These facts mean that more than 50% of creep deformation can be represented as a steady state in the wide range of applied stress.

## 5. Conclusion

We calculated the Bayesian free energy for the modified theta projection with a steady-state term and the theta projection for a Gr.91 steel under various testing conditions in the ranges of 50–90 MPa at 923 K, 90–160 MPa at 873 K and 170–240 MPa at 823 K. The Bayesian free energy was clearly lower for the modified theta projection under all the conditions and the calculated posterior probability was virtually 1.0. We thus concluded that the experimental data support the modified theta projection. Detailed analysis of the strain rate versus time plot showed that more than 50% of the creep deformation can be regarded as occurring in the steady state. The double logarithmic plot of the steady-state creep rate versus applied stress indicated that there are two stress regions in terms of the mechanism governing the steady state below and above ~120 MPa.

The present study demonstrated that the Bayesian inference framework is useful to rationally judge what type of the constitutive equation should be applied in creep deformation. We consider that this framework may lead to a useful insight not only for the creep deformation but also for any other fields in materials science. For applying the framework, each model in a targeted controversial issue should be formulated. In addition, it would be necessary to modify the framework itself so as to handle all the model parameters as probabilistic ones, that is, a fully Bayesian inference framework would be crucial in some non-linear formulations.

## Supplementary Material

Supplemental Material
