# Supplementary material for: Data-based selection of creep constitutive models for high-Cr heat-resistant steel
Source: Sci Technol Adv Mater. 2020 Apr 27;21(1):219–28. doi: 10.1080/14686996.2020.1738268 (PMC7241477; doi:10.1080/14686996.2020.1738268)
Supplement: Supplemental Material [file TSTA_A_1738268_SM3722.pdf]

Data-based selection of creep constitutive models for high-Cr heat-resistant steel

Hitoshi Izuno[a], Masahiko Demura[a]*, Masaaki Tabuchi[b], Yoh-ichi Mototake[c], Masato Okada[a,c]

[a] *Research and Services Division of Materials Data and Integrated System, National Institute for Materials Science, Namiki 1-1, Tsukuba, Ibaraki 305-0044, Japan*

[b] *Research Center for Structural Materials, National Institute for Materials Science, Sengen 1-2-1, Tsukuba, Ibaraki 305-0047, Japan*

[c] *Graduate School of Frontier Sciences, The University of Tokyo, Kashiwa, Chiba 277-8561, Japan*

Information Classification: General

Figure S1. Log of the creep strain rate against time with its fitting by modified theta method for all test condition, except for 823 K/240 MPa. Blue broken, red dot-dashed, and green dotted lines for each plot are the asymptotes of the corresponding terms of the equation. The gray horizontal line shows the region where the steady-state term (equation (12)) is more than 10 times larger than the other terms (equations (11) and (13)).

Information Classification: General

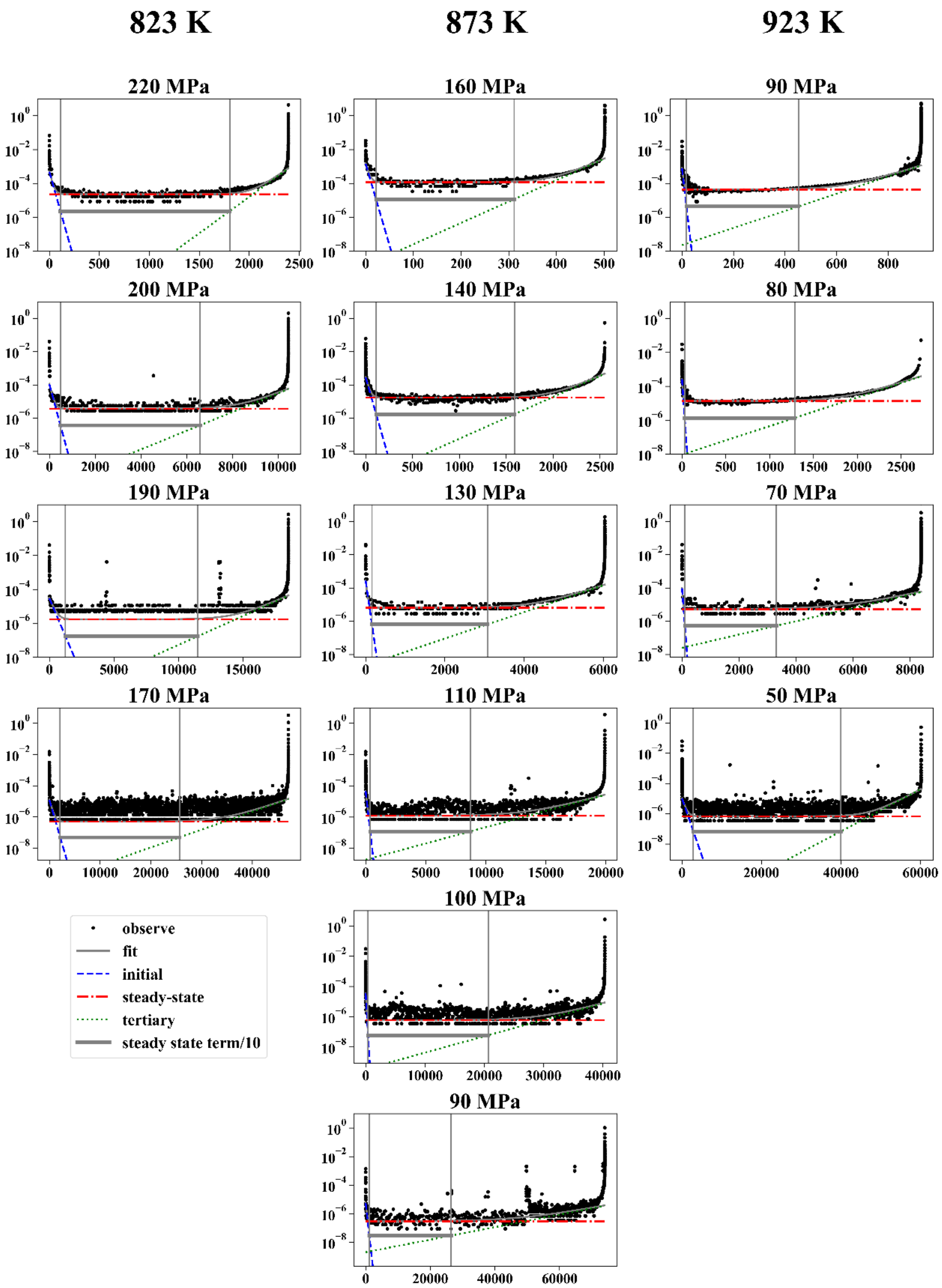

Information Classification: General
